# Isolation and Characterization of Specific Phages to Prepare a Cocktail Preventing *Vibrio* sp. Va-F3 Infections in Shrimp (*Litopenaeus vannamei*)

**DOI:** 10.3389/fmicb.2019.02337

**Published:** 2019-10-11

**Authors:** Ling Chen, Jiqiang Fan, Tingwei Yan, Quan Liu, Shengjian Yuan, Haoran Zhang, Jinfang Yang, Deng Deng, Shuqiang Huang, Yingfei Ma

**Affiliations:** ^1^Institute of Synthetic Biology, Shenzhen Institutes of Advanced Technology, Chinese Academy of Sciences, Shenzhen, China; ^2^Key Laboratory of Quantitative Engineering Biology, Chinese Academy of Sciences, Shenzhen, China; ^3^Shenzhen Key Laboratory of Synthetic Genomics, Shenzhen Institutes of Advanced Technology, Chinese Academy of Sciences, Shenzhen, China; ^4^Guangdong Provincial Key Laboratory of Genome Read and Write, Shenzhen Institutes of Advanced Technology, Chinese Academy of Sciences, Shenzhen, China; ^5^College of Life Science and Technology, Jinan University, Guangzhou, China; ^6^College of Life Science and Oceanography, Shenzhen University, Shenzhen, China; ^7^University of Chinese Academy of Sciences, Beijing, China; ^8^R&D Center, Shenzhen Alpha Feed Co., Ltd, Shenzhen, China

**Keywords:** vibriosis, phage therapy, *Vibrio*, phage cocktail, shrimp

## Abstract

*Vibrio* is one of the most detrimental agents of shrimp premature death syndrome. Phage therapy for prevention and treatment of *Vibrio* infections has attracted increasing attentions due to the emergence of antibiotic-resistant bacterial variants. Here, we describe a workflow of preparing a phage cocktail against *Vibrio* infections for practical applications. Twenty *Vibrio* strains were isolated from the gut of diseased shrimp and aquaculture wastewater, and five of them were identified as pathogens causing shrimp vibriosis. Twenty-two lytic phages were then isolated using the above five pathogens as hosts, and five of them showed broad host ranges and high lytic capability against the *Vibrio* strains. Whole genomic sequencing and phylogenetic analysis of the five phages indicated that they are novel and belong to the *Siphoviridae* family. The phage cocktail consisting of these five phages showed higher efficiency in inhibiting the growth of pathogenic *Vibrio* sp. Va-F3 than any single phage *in vitro*. We then evaluated the performance of the phage cocktail in protecting shrimp against *Vibrio* sp. Va-F3 infections *in situ*. The results showed that shrimp survival rates could reach 91.4 and 91.6% in 7 days, for the cocktail-treated and the antibiotic-treated groups, respectively. By contrast, the shrimp survival rate of the group without any treatment was only 20.0%. Overall, this study describes a general workflow of how to prepare a phage cocktail and apply it in controlling bacterial infections in the shrimp aquaculture. Knowledge gained from this study will not only help fight against the shrimp vibriosis in practical but also facilitate the design of phage cocktails with a satisfying performance in controlling other animal diseases in aquaculture.

## Introduction

*Vibrio* is a genus of Gram-negative, comma-shaped rod bacteria and grows preferentially in warm (>15°C) marine water (Bakeraustin et al., 2010) with low salinity (<25 ppt NaCl). In shrimp aquaculture, the severe infections of various *Vibrio* strains often cause the shrimp disease of vibriosis (Frans et al., 2011). Traditionally, various antibiotics (e.g., sulfonamides and tetracyclines) have been widely used in the treatments of the infections of *V. parahaemolyticus*, *V. harveyi*, *V. alginolyticus*, and other *Vibrio* strains in the shrimp aquaculture (Tendencia and de la Peña, 2001; Vinod et al., 2006; Al-Othrubi et al., 2014; Wang et al., 2015), consequently, causing the alarming emergence of multidrug-resistant pathogens. It is imperative to develop alternative strategies to prevent and treat the shrimp diseases caused by drug-resistant pathogenic bacteria infections (Miller and Miller, 2011; Young and Gill, 2015).

Bacteriophage was first discovered in 1915 by Twort (1972) and D’Herelle (2011). Since then, bacteriophage has been used to treat bacterial infections (commonly termed phage therapy). Nowadays, there has been a renewed interest in applying phage therapy to control bacterial pathogens due to the limited use of antibiotics (Golkar et al., 2014). So far, phage therapy has been applied in controlling the bacterial infections of plants, humans, domestic animals, and marine animals as well as a biological control for food productions (Chan et al., 2013; Doss et al., 2017; Kalatzis et al., 2018). The use of phage therapy has been proven to be medically safe and effective in several cases (Biswas et al., 2002; Karunasagar et al., 2007; Chhibber et al., 2009; Chadha et al., 2016; Wang et al., 2017).

To date, several phages against *V. alginolyticus* have been isolated and reported, including two myoviruses PVA1 (Zhang et al., 2014) and phi-A318 (Liu et al., 2014) and two podoviruses ValKK3 (Lal et al., 2016) and VEN (Kokkari et al., 2018). However, direct use of single phage for combating bacterial infections is quite limited because of low efficacy, narrow host range of single phage, and fast emergence of phage-resistant bacterial mutants (Pirnay et al., 2011; Chan et al., 2013; Letchumanan et al., 2016; Yen et al., 2017; Kilcher et al., 2018). Our study aims to explore the potential use of phage therapy as antibacterial tools in aquaculture against bacterial infections to combat the antibiotic crisis. Taking the disease of vibriosis as an example, here we describe an integrated workflow of how to develop an appropriate phage cocktail against the *Vibrio* pathogens and then determine the effectiveness of the cocktail in treating shrimp diseases caused by *Vibrio* sp. Va-F3 infections *in situ*.

## Materials and Methods

### Animals and Conditions

Healthy two-month-old shrimp (*L. vannamei*; weight = 10.2 ± 0.6 g) and the commercial feeds for shrimp were provided by Aolong Seedling Plant of Shenzhen Alpha Feed Agriculture and Animal Husbandry Co., Ltd., Shanwei city, China (N: 22.782051, E: 115.541900). The shrimp were placed and cultured at Aolong Seedling Plant. The aquatic water temperature was maintained at 24 ± 1°C during the study.

### Isolation and Identification of *Vibrio* Pathogens

*Vibrio* pathogenic strains used in this study were isolated from both the gut of the diseased shrimp collected from aquaculture pools where numerous shrimp died of vibriosis and the aquaculture wastewater samples collected from the drain exits of shrimp culturing pools (Nov. 2016, Aolong Seedling Plant, Shanwei city, China). The *Vibrio* strains were isolated by the selective medium containing thiosulfate citrate bile salt sucrose agar (TCBS agar, Haibo, China) and further incubated at 28°C in 2216E medium (5 g peptone and 2 g yeast extract per liter; Wang et al., 2015). Taxonomic assignment of these purified strains was performed based on the analysis of 16S rRNA gene sequences (Frank et al., 2008). Briefly, microbial DNA samples were used as the substrate for amplification of 16S rRNA gene fragments using the universal primers 27f and 1494r (Xu et al., 2018). The reaction mixture consists of 10 ng of template DNA, 10 pmol of each primer, 2.5 U of DNA polymerase, 5 μl of 10× PCR amplification buffer (100 mM Tris-HCl and 500 mM KCl), 200 μM dNTP, 1.5 mM MgCl_2_, and 10 pmol of a primer. The above mixture was first denatured for 1 min at 98°C, followed by 30 typical PCR cycles of denaturation (10 s at 98°C), annealing (30 s at 56°C), and extension (40 s at 72°C). Finally, another extension was executed at 72°C for 2 min. The PCR products were sequenced by Sanger sequencing platform (BGI gene Co., Ltd., China). The 16S rRNA gene sequences have been submitted to the NCBI GenBank database with accession numbers (Supplementary Table S1).

The verified pathogenic strains were further typed based on the method described previously (Szczuka and Kaznowski, 2004). Briefly, ERIC-PCR (enterobacterial repetitive intergenic consensus sequence PCR) method with primers ERIC-R (5′-ATGTAAGCTCCTGGGGATTCAC-3′) and ERIC2 (5′-AAGTAAGTGACTGGGGTGAGCG-3′) (Versalovic et al., 1991) was employed to type the subspecies of the strains. The reaction mixture was denatured for 7 min at 95°C, then subjected to 30 cycles of denaturation for 30 s at 90°C, annealing for 1 min at 52°C, extension for 8 min at 65°C, and a final extension for 16 min at 65°C. The patterns of PCR products were visualized by 1.5% agarose gel (wt/vol) in 1× Tris-acetate buffer (40 mM Tris-acetate, 1 mM EDTA) running for 40 min under 80 V.

To determine the pathogenicity of the isolated *Vibrio* strains, healthy shrimp were challenged with the isolated *Vibrio* strains in April 2017. In total, 750 healthy shrimp were randomly divided into 25 groups, with 30 shrimp per group. For the control group, none of the *Vibrio* strains was included, whereas for the challenge groups, 500 ml of the bacterial suspension of each tested strain was added to the aquatic water at different concentrations (2.2 × 10^2^, 2.2 × 10^4^, and 2.2 × 10^6^ CFU/ml). Each group of the shrimp was raised within a 20 L plastic bucket. The survival rate of the shrimp of each group was recorded after 6 days. Low survival rate of the shrimp suggests that the corresponding *Vibrio* strain used for challenge experiment is likely the pathogen of vibriosis.

### Isolation and Purification of Bacteriophages

The verified pathogens from above experiment were then used as hosts to isolate their specific phages. The phages were isolated from the wastewater samples collected from sewage draining exits in the cities of Shenzhen, Zhanjiang, and Shanwei, China, respectively. The wastewater samples were collected in April-November 2017 and April-July 2018. For each sample, approximately 20–50 ml was collected into sterile homogeneous bags (500 ml, Hopebiol) and stored at 4°C. The samples were centrifuged at 8,000× *g* for 10 min, and the supernatants were filtered through a 0.22-μm pore-size membrane to remove the solid impurities and bacterial cells. The filtrates were then mixed with 1× 2216E medium and the pathogenic strain cultures for phage enrichment at 28°C overnight. Subsequently, the cultures were centrifuged again at 8,000× *g* for 10 min, and the supernatants were then filtered through the 0.22-μm pore-size membrane. The bacteriophage titer of the filtrate was determined using the double-layered method (Chen et al., 2018). Clear phage plaques were picked from the plates and placed into 1 ml of sterile 2216E medium. This separation procedure was repeated three times to ensure the purity of the isolated phages.

### Determination of Phage Host Ranges

In total, 20 *Vibrio* strains (isolated from the above section of *isolation and identification of the bacterial pathogens*) were included to determine the host ranges of the isolated phages (Supplementary Table S1). Lytic capabilities of the isolated phages were evaluated using standard spot tests. Briefly, the bacterial strains were mixed with 0.7% 2216E medium top agar and overlaid on 1.5% 2216E medium plates, and then 10 μl of the purified phage suspension (10^8^ PFU/ml) was dropped in the middle of each plate. The plates were examined in 12 h after incubation at 28°C. The bacterial hosts of a tested phage were confirmed if a clear phage lytic plague could be observed in the plate. This procedure was replicated three times for verification.

### Transmission Electron Microscopy

Phage particles were precipitated with 10% polyethylene glycol 8,000 (PEG 8000) at 4°C overnight, centrifuged at 10,000× *g* for 15 min, and subsequently suspended in SM buffer (100 mM NaCl, 8 mM MgSO4, 50 mM Tris-HCl, and 0.01% gelatin). One drop of the concentrated phage particles was placed on copper grids with carbon-coated formvar films, followed by negative staining with 4 μl of 2% (wt/vol) phosphotungstic acid (pH 6.5). Then, the grids were dried and examined using a Tecnai G2 F20 S-Twin electron microscope (FEI, USA) operated at 120 kV of accelerating voltage.

### Phage DNA Extraction, Genome Sequencing, and Assembly

The concentrated phage particles were treated using DNase I and RNase A (New England BioLab, England) to remove bacterial nucleic acids. Then, phage genomic DNA was extracted using the Lambda Bacteriophage Genomic DNA Rapid Extraction Kit (Aidlab, China) following the manufacturer’s protocol. The purified phage DNA was sequenced using the Illumina HiSeq1500 sequencer platform (Annoroad gene technology Co. Ltd., China). The filtered reads were assembled using SOAP *de novo* by the default parameters (Luo et al., 2012). The complete genome of each phage was finished and then manually inspected.

### Genome Analysis and Phylogenetic Analysis

Open reading frames (ORFs) encoded by the complete phage genomes were predicted by the program GeneMark.hmm (Besemer and Borodovsky, 2005). The ORFs were annotated using the BLASTP algorithm against the non-redundant (nr) protein database of the National Center for Biotechnology Information (NCBI; Song et al., 2009; Sharma et al., 2016) in January 2019. The putative promoter sequences and regions on the phage genomes were identified using promoter online analysis tools of Softberry (Shahmuradov et al., 2003) and Promoter Scan (Prestridge, 2000). We tried to detect virulent genes on the phage genomes using the Virulence Search program (Underwood et al., 2005). Rho-independent transcription terminators on the phage genomes were also predicted using the ARNold program (Naville et al., 2011). tRNAs carried by the phage genomes were detected using the protein analysis software of ARAGORN and tRNAscan-SE (Schattner et al., 2005). Comparative genome analysis of the isolated phages was conducted using EasyFig 2.1 (Rombouts et al., 2016). To determine the taxonomy of the isolated phages, phylogenetic analysis of the phages based on the protein sequences of large terminase subunits was carried out using the MEGA 5.02 software with the neighbor-joining method and 1,000 bootstrap replications (Tamura et al., 2011). Comparison of pairwise similarity of phage genomes based on their predicted proteins was performed using BLASTP and visualized using R (version 3.4.1) gplots package (Walter et al., 2015).

### Inhibition of Pathogen by Phages *in vitro*

*Vibrio* sp. Va-F3 was selected as the host for phage inhibition test. Phages were selected for phage cocktail design based on the criteria: (1) the phages have broad and different host ranges; (2) the phages were isolated from the samples with different locations, and to the most degree, this warrants that the isolated phages are different without the knowledge of the genome sequences; and (3) the host ranges of the five phages cover the highest number of the tested bacteria (Crothers-Stomps et al., 2010; Chan et al., 2013). The phage cocktail was prepared by pooling equal volume of each purified phage solution at a concentration of 10^9^ PFU/ml. Bacterium Va-F3 grew to the optical density at 600 nm (OD_600_) of approximately 0.5, which was equal to approximately 10^8^ CFU/ml. Each well of a 96-well plate was loaded with 100 μl bacterial culture and 100 μl of dilutions of the five phages and the cocktail (MOI, multiplicity of infection = 10). Three replications of each test were performed as well. Plate sterility, bacterial culture without any phage, and phage suspension without any bacterial cell were set up as controls. All the plates were incubated at 28°C for 36 h, and the value of OD_600_ of each plate was measured using the Bioscreen plate reader (Bioscreen C, Finland) at 30 min intervals.

### Pathogenic Infections of Shrimp by *Vibrio* sp. Va-F3 and Phage Therapy *in situ*

To validate the therapeutic performance of the designed cocktail in controlling the pathogenic bacterial infections in practical applications, 540 healthy shrimp (weight = 10.2 ± 0.6 g) were equally divided into six groups for the experiment in November 2017. Each group contained 90 shrimp and was then divided equally into three parallel subgroups as replicates (30 shrimp per subgroup). Group I was supplemented with fresh 2,216 medium only. Group II was challenged with *Vibrio* sp. Va-F3 within a culturing bath at a concentration of 2 × 10^6^ CFU/ml. Group III was treated with 10 mg/L kanamycin after a two-day challenge of Va-F3. Group IV was supplemented with the phage cocktail at a final concentration of 2 × 10^7^ PFU/ml but without the challenge of Va-F3. Group V received the treatment of the phage cocktail at a final concentration of 2 × 10^7^ PFU/ml after a two-day challenge of strain Va-F3 at 2 × 10^6^ CFU/ml. The control group was set as the one without any treatment. The survival rate of the shrimp of each group was subsequently summarized after 7-day cultivation.

### Nucleotide Sequence Accession Number and Phage Preservation

The phages were preserved in China Center for Type Culture Collection (Wuhan, China) with CCTCC Nos. M2107548 of ValLY-3, M2107549 of VspDsh-1, M2107547 of VpaJT-1, M2107552 of ValSw4-1, and M2107551 of VspSw-1. The complete genome sequences of the phages have been submitted to the NCBI GenBank database with accession numbers: MH925090 of ValLY-3, MH925091 of ValSw4-1, MH925092 of VpaJT-1, MH925093 of VspDsh-1, and MH925094 of VspSw-1.

## Results

### Isolation and Characterization of *Vibrio* Pathogens

**Figure 1:**
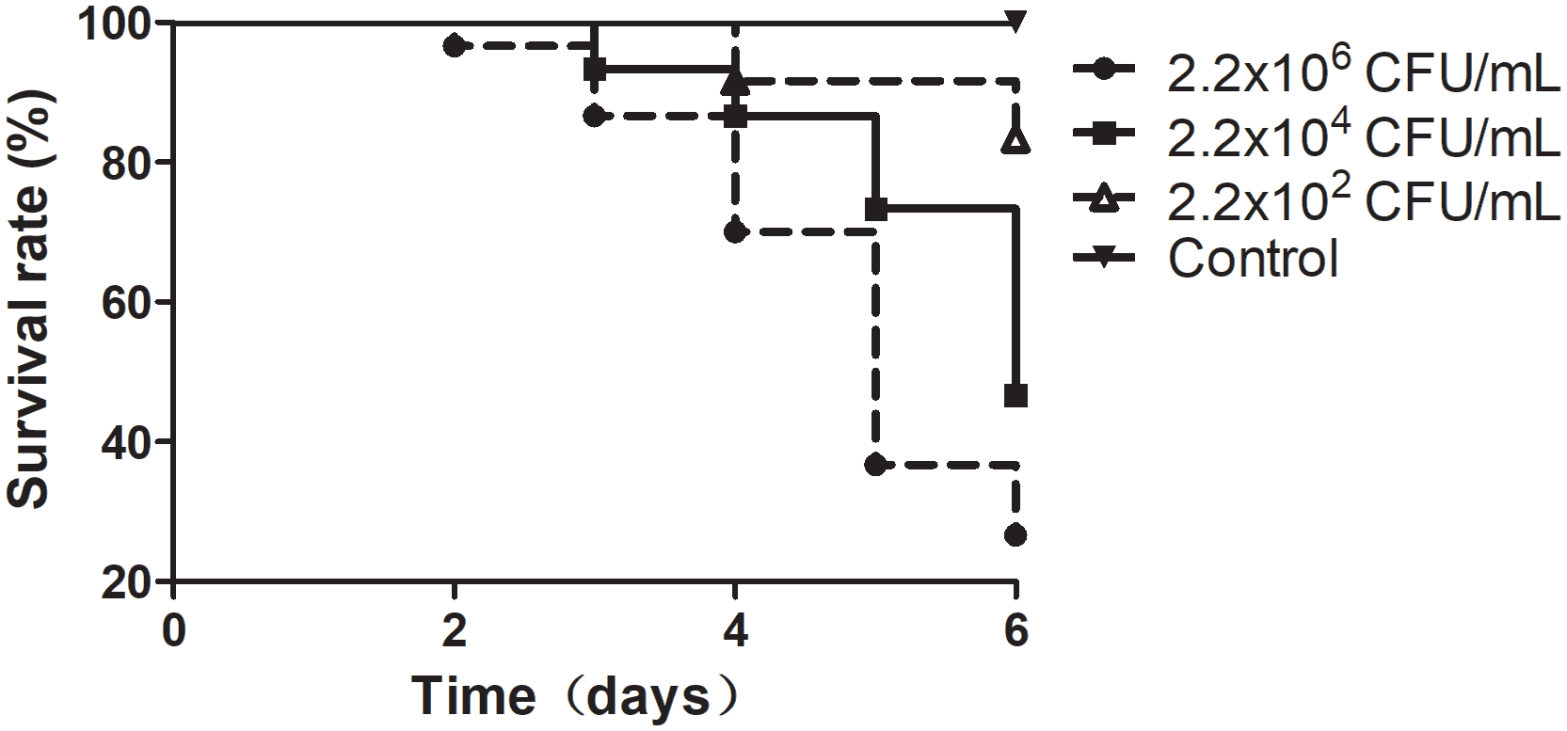
Survival rates of the shrimp infected by *Vibrio* sp. Va-F3. Survival rates of the shrimp were calculated after cocultured with *Vibrio* strains for 1 week. The control group was set as no *Vibrio* strain included, and the experimental groups included 500 ml of bacterial suspension with three different bacterial concentrations, as 2.2 × 10^2^, 2.2 × 10^4^, and 2.2 × 10^6^ CFU/ml.

In this study, in total, 20 *Vibrio* strains were successfully identified, and the sequences of their 16S rRNA genes have been submitted to the NCBI GenBank database (Supplementary Table S1). According to the top hits of their 16S rRNA genes in NCBI nr database, 1 strain has top hit to *V. ovensii*, 8 to *V. parahaemolyticus*, 2 to *V. natriegens*, 2 to *V. metschnikovii*, 2 to *V. azureus*, and 5 to *V. alginolyticus*. These 20 *Vibrio* strains were then used to challenge the healthy shrimp to determine if they can cause shrimp vibriosis. Five *Vibrio* sp. strains Va-F4, Va-F2, Va-F3, Va-F10, and Val-3 are likely the pathogens of shrimp vibriosis because the mortality rates of the shrimp challenged by these strains reached from 16.7 to 73.4% after 6-day cultivation (Figure 1; Supplementary Figure S1; Supplementary Table S1). Meanwhile, we further typed the subspecies of the five *Vibrio* strains using ERIC-PCR primers, and the results indeed showed slightly different patterns of their PCR products (Supplementary Figure S2), suggesting that these five strains belong to different subtypes of the same species within the genus of *Vibrio*. Besides, among the five pathogenic strains, Va-F3 showed the highest pathogenic capability to the shrimp (73.4% mortality rate as shown in Figure 1). Therefore, Va-F3 was selected as the representative pathogen for the following studies.

### Isolation of Lytic Bacteriophages Using Pathogenic *Vibrio* Strains as Hosts

**Figure 2:**
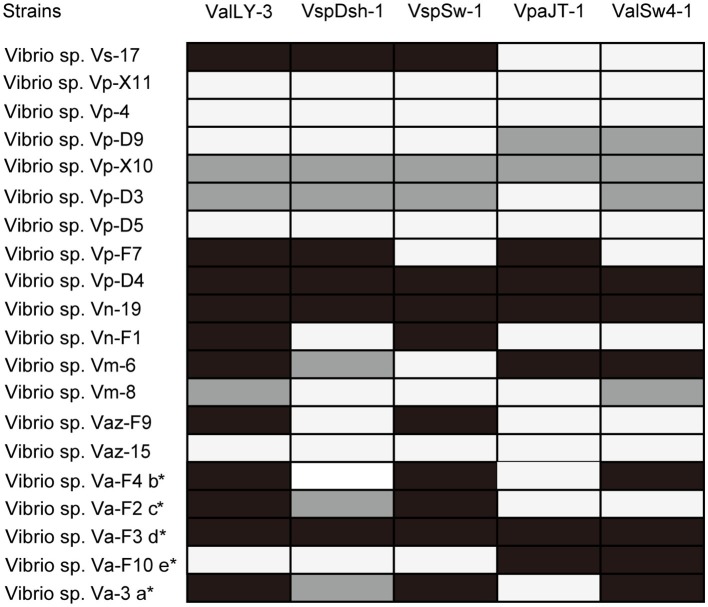
Host ranges of phages ValLY-3, VspDsh-1, VspSw-1, VpaJT-1, and ValSw4-1. A total of 20 bacterial strains were used for determining the phage host ranges. The 16S rRNA gene sequences of all the strains have been sequenced and submitted to the NCBI with accession as attached above (more information referring to Supplementary Table S1). Black spots indicated clear plaque, gray for turbid plaque, and white for no phage plaque. a: The host strain used for VspDsh-1 isolation; b: VspSw-1 isolation; c: VpaJT-1 isolation; d: ValLY-3 isolation; e: ValSw4-1 isolation. ^*^, Verified pathogenic strains in this study.

Using the five pathogenic *Vibrio* strains as hosts, we successfully isolated 22 bacteriophages from the collected water samples. These phages can form clear plaques on their host strains following overnight incubation at 28°C, suggesting that all these phages are likely lytic phages against their hosts. The host ranges of the isolated phages were then determined using the 20 *Vibrio* strains isolated in this work (Figure 2; Supplementary Table S1). According to the results, five phages that were named ValLY-3, VspDsh-1, VspSw-1, VpaJT-1, and ValSw4-1 have broader host ranges against *Vibrio* strains, respectively (Figure 2). Notably, phage ValLY-3 appeared to be more effective against *Vibrio* sp. strains because it could lyse four of the five pathogenic *Vibrio* sp. strains and the top hits of their 16S rRNA genes in NCBI nr database all belong to *V. alginolyticus* (Supplementary Table S1). However, none of the isolated phages could individually infect all the five pathogens (Va-F4, Va-F2, Va-F3, Va-F10, and Val-3). Based on the observation that the host range of combining the five phages covers all the five pathogenic strains, it is likely that the cocktail consisting of these five phages can be applicable to effectively inhibit the growth of the *Vibrio* pathogens causing vibriosis *in situ*.

### Morphology of the Lytic Bacteriophages

**Figure 3:**
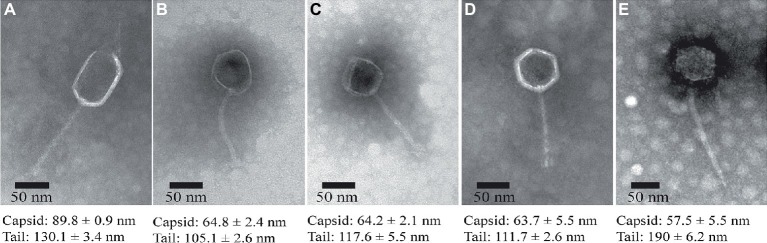
TEM images of the isolated lytic phages. **(A)** ValLY-3, **(B)** VspDsh-1, **(C)** VpaJT-1, **(D)** ValSw4-1, and **(E)** VspSw-1. The phages were stained with 2% phosphotungstic acid and visualized at 120,000× magnification with transmission electron microscopy. Scale bars represent 20 nm.

The morphologies of the isolated phages were observed using TEM, indicating that all the five phages exhibit hexagonal heads (diameter ranged from 55 to 90 nm) and non-contractile, flexible tails (length ranged from 100 to 200 nm), which are typical features of the phages belonging to the *Siphoviridae* family (Figure 3), although ValLY-3 and ValSw4-1 have a larger size in head with the diameters of 89.8 and 57.5 nm, respectively.

### Genome Sequencing and Annotation of the Phages

**Figure 4:**
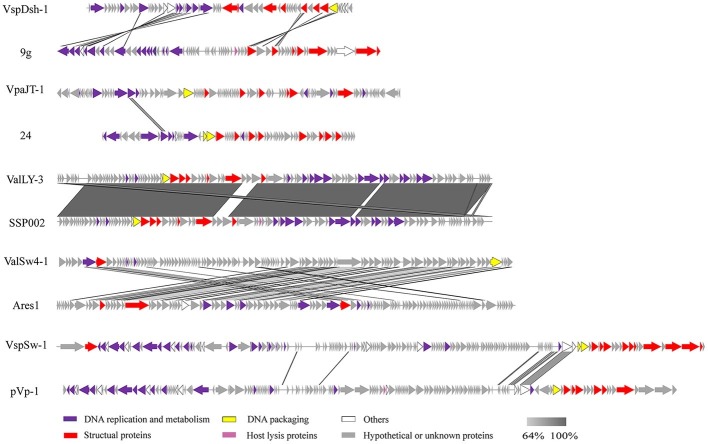
Multiple sequence alignment of phage genomes. Whole genome sequences of the isolated phages (ValLY-3, VspDsh-1, VpaJT-1, ValSw4-1, and VspSw-1) and the highest similar reference phages were compared using Easyfig. The gray regions indicate high similarity among the genomic sequences. The predicted functional proteins are indicated by different colors. Blastn analyzes of the five phages’ genomes show that the top hits in NCBI nr database of ValLY-3, VspDsh-1, VpaJT-1, ValSw4-1, and VspSw-1 are *Vibrio* phage SSP002 (JQ692107.1, with cover 95% and identity 99%), *Enterobacteria* phage 9 g (KJ419279.1, with cover 0% and identity 80%), vB_SenS_Sergei (KY002061.1, with cover 0% and identity 100%), *Vibrio* phage Ares1 (MG720309.1, with cover 31% and identity 67%), and *Vibrio* phage pVp-1 (JQ340389.1, with cover 2% and identity 79%), respectively.

We further sequenced and characterized the whole genome sequences of the five phages. The features of the genomes were summarized in Table 1. The genomes range in size from 46.6 to 113.7 kb with GC contents of 43.8–49.2%. The GC contents of the five phages are lower than those of their *Vibrio* host genomes (averagely, 50%). Phage VspSw-1 encodes the highest number (*n* = 25) of tRNAs. By contrast, the other phages encode few tRNAs (≦2), indicating that they mainly depend on the host translation machinery. ORFs encoded by the phage genomes were predicted, and the corresponding functions were annotated by Blastp analysis against the NCBI nr database. About 66.2% (351/530) of ORFs share 26–100% identities of amino acid sequences with those deposited in the NCBI GenBank database. We identified the ORFs that were assigned to the basic phage-related functions (Figure 4), including DNA replication, DNA metabolism, DNA packaging, structural proteins, and host lysis. Neither the genes responsible for toxins or virulence in the ARDB database (Liu and Pop, 2008) or the VFDB database (Chen et al., 2005), nor the known lysogenic-related genes (Oppenheim et al., 2005) were found within the genomes of the five phages. These genetic features ensure that these phages are suitable candidates for phage therapy.

**Table 1:** Genomic features of the five phages.

		Phage name				
		VspDsh-1	VpaJT-1	ValLY-3	ValSw4–1	VspSw-1
Features	Genome length (bp)	46,692	60,177	76,310	79,545	113,778
	GC content (%)	46.7	49.2	48.8	45.7	43.8
	ORFs	60	96	104	112	158
	Terminators	25	29	57	58	68
	Promoters	110	134	179	190	271
	tRNAs	1	2	0	0	25
	Host	*Vibrio* sp. Va-F4	*Vibrio* sp. Va-F3	*Vibrio* sp. Val-3	*Vibrio* sp. Va-F3	*Vibrio* sp. Va-F2
	Accession ID	MH925093	MH925092	MH925090	MH925091	MH925094

**Figure 5:**
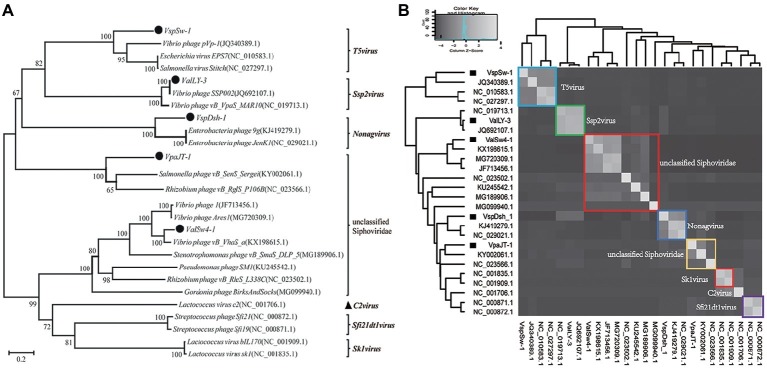
Phylogenetic analysis of the five isolated bacteriophages. **(A)** Phylogenetic tree based on the sequences of the large subunits of terminases of the selected bacteriophages. The sequences of the large subunits of terminases were aligned using the Mega 5.05 program, and the phylogenetic tree was generated using the neighbor-joining method with 1,000 bootstrap replications. **(B)** Heat map showing percentage of the shared protein sequences among the bacteriophages.

Whole genomic sequence alignment also demonstrates the novelty of the five phages at the genomic levels. As shown in Figure 4, the phages have only few cross wires with those in the NCBI GenBank database, except for ValLY-3, whose genome highly resembles that of previously characterized *Vibrio* phages *SSP002* (JQ692107.1), with the identity of 95% (Table 1). Phylogenetic analysis was performed based on the large terminase subunits identified in ValLY-3, ValSw4-1, VpaJT-1, VspDsh-1, and VspSw-1, as well as other 21 classified phages ratified by ICTV (Figure 5A). The results indicated that the five phages belong to five different genera within the *Siphoviridae* family. VpaJT-1 and ValSw4-1 are members of two different unclassified genera within the *Siphoviridae* family, respectively, as they are grouped into different clades in the phylogenetic tree (Figure 5A). Moreover, we also conducted an *in silico* pairwise comparison of the proteomes of the phages used in the phylogenetic analysis. Eight different groups can be clustered, in line with the result of the phylogenetic analysis (Figure 5B). This analysis suggests that the five phages are different and likely employ different mechanisms to infect the *Vibrio* hosts.

### Inhibition of Bacterial Growth by Phages *in vitro*

**Figure 6:**
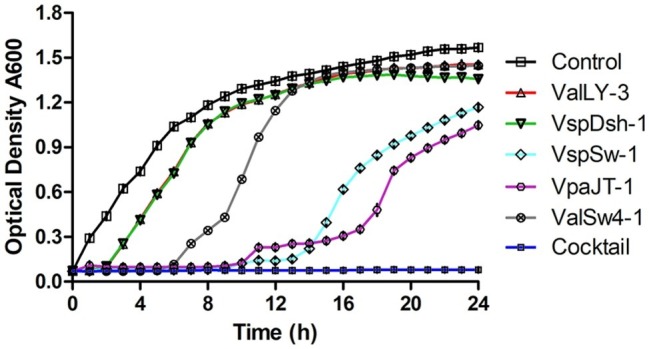
Growth inhibition of *Vibrio* sp. Va-F3 by the five phages and the phage cocktail *in vitro*. The MOI was set as 10, the optical density of bacterial solution as approximately 0.5 (OD_600_, 10^8^ CFU/ml), and the concentrations of the phages (ValLY-3, VspDsh-1, VspSw-1, VpaJT-1, ValSw4-1, and their cocktail) as 2 × 10^9^ PFU/ml. Each point represents the average result of three replicates, and error bars represent standard deviations. According to the curve, we can calculate the frequency of phage resistant bacteria in the population to a given phage. First, we calculated the number of the phage resistant bacterial cells starting to grow during the incubation using the formula: Nm = Nt(the number of bacterial cells at OD_600_ = 1)/2^[tOD600(the time bacteria grow to OD600 = 1)/td(bacterial cell doubling time)]^, and then f(the frequency of phage resistant bacteria) = Nm(the number of bacterial mutant cells at beginning)/N0(the number of all bacterial cells at beginning). We determined N0 = 2 × 10^8^ CFU/ml and td = 60 min; at OD_600_ = 1, tOD_600_ = 5 h for control, tOD_600_ = 8 h for ValLY-3 and VspDsh-1, tOD_600_ = 11 h for ValSw4-1, tOD_600_ = 20 h for VspSw-1, and tOD_600_ = 23 h for VpaJT-1; Nt = 10^9^. Therefore, we calculated the f(ValLy-3 and VspDsh-1) = 0.20, f(ValSw4-1) = 0.002, f(VspSw-1) = 4.77E-06, f(VpaJT-1) = 5.96E-07. During the experiment in 24 h, we were not able to determine the growth of the bacterial cells when treated with the cocktail, suggesting the frequency of the cocktail resistant bacterial cells is extremely low.

We then tested the infective capability of the five phages and the cocktail containing five phages against *Vibrio* sp. Va-F3 *in vitro*, respectively. Strain Va-F3 was verified to be the pathogen with the highest mortality rate against the shrimp in our study (Figure 1; Supplementary Figure S1). As shown in Figure 6, all the five phages were found to be capable of inhibiting the growth of Va-F3, but the host growth recovery started in 2, 2, 6, 9, and 9 h when treated with phages VspDsh-1, ValLY-3, ValSw-1, VpaJT-1, and VspSw-1, respectively. This suggests different abilities of the five phages in inhibiting the growth of Va-F3. For the control group without phage treatment, the OD_600_ value of strain Va-F3 reached 0.45 after 2 h incubation, while the growth of Va-F3 was inhibited in all phage-treated groups within the first 2 h. By contrast, the inhibition effectiveness of the cocktail was higher than those of any single phage, as we can see that the growth inhibition of the host in the cocktail-treated group lasted for at least 24 h (Figure 6).

### Application of the Cocktail Against *Vibrio* sp. Va-F3 Infections of the Shrimp *in situ*

**Figure 7:**
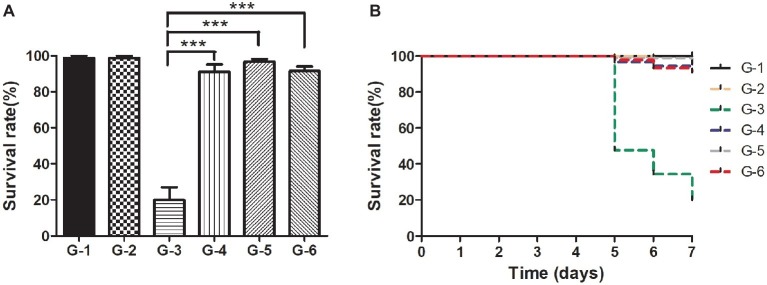
Comparison of the survival rates of the shrimp in different treatment groups *in situ*. **(A)** Survival rates of the shrimp of each group after the 7-day treatment; **(B)** Survival rates of each group in the experiment duration. C: The control group was set up without any treatment. G-1: Group I was given fresh 2,216 medium only. G-2: Group II was challenged with *Vibrio* sp. Va-F3 at concentration of 2 × 10^6^ CFU/ml. G-3: Group III was treated with 10 mg/l kanamycin after two-day challenge of Va-F3 at 2 × 10^6^ CFU/ml. G-4: Group IV received treatment of the phage cocktail at the final concentration of 2 × 10^7^ PFU/ml. G-5 Group V received treatment of the phage cocktail at the final concentration of 2 × 10^7^ PFU/ml after two-day challenge of strain Va-F3 at 2 × 10^6^ CFU/ml. Asterisks above the columns indicate significant differences at the *p* < 0.01 level (Wilcoxon-Mann-Whitney test).

For practical application of phage therapy, we sought to validate the performance of the cocktail against the pathogenic infections of the shrimp *in situ*. In this experiment, we set up one control group and five experimental groups. The shrimp of group II were infected by the pathogen without any treatment, and the skin color of the shrimp became white and turbid and the intestine turned to be slightly red in 5 days. Meanwhile, the body of the infected shrimp was soft, the size of their hepatopancreas was enlarged, and the color was light yellow (Supplementary Figures S3A–D). The survival rate of the shrimp of group II reached only 20% in 7 days (Figures 7A,B). In comparison, the survival rate of the shrimp of group V treated by the cocktail reached 91.4%, which was quite comparable to that of group III treated by antibiotics (91.1%, Figure 7A). Furthermore, to eliminate the concern of potential phage toxicity, we included group IV where the shrimp got the cocktail treatment only, and the survival rate was similar to those of groups III and V, suggesting that no apparent side effects of the phages were detected.

## Discussion and Conclusion

The concept of using phages as an agent for controlling pathogen infections has been widely accepted. There have been numerous studies describing the applications of phage therapy in controlling pathogen infections in aquaculture (Rao and Lalitha, 2014). However, the application of phage therapy in aquaculture as well as in clinics is still greatly limited. This can be attributed to the lack of a standard workflow of how to prepare a phage cocktail for controlling pathogen infections in practical applications. To our knowledge, for the first time, our study describes the integrated workflow starting from step (1) isolation and verification of the Vibrio pathogens from diseased animals; step (2) specific phage isolation, sequencing, and characterization; and step (3) phage cocktail preparation for the efficiency test *in vivo* and *in situ*. Each step of the workflow is necessary and will be the warrant of developing a safe and efficient phage cocktail in an application. Some studies directly used the pathogens provided by other studies for phage isolation and phage cocktail preparation (Higuera et al., 2013; Kalatzis et al., 2016). Thus, these bacterial strains may not be able to cause vibriosis *in situ*. Meanwhile, some studies failed to obtain the sequences of the phages used for phage cocktail (Higuera et al., 2013; Mateus et al., 2014; Zhang et al., 2015). This sequencing information of the phages could be quite helpful, and thus, we may exclude those phages carrying toxin genes for phage therapy based on the bioinformatics analysis of their gene sequences before phage cocktail preparation. More importantly, in our study, the *in situ* experiment showed that the administration of the resulted phage cocktail in treating the *Vibrio* infections of the shrimp promoted the survival rate of the shrimp as significantly as the antibiotic treatment (Figure 7).

Instead of using the *Vibrio* pathogens reported in other literature (Mechri et al., 2017; Rameshkumar et al., 2017; Lv et al., 2019) as hosts for lytic phage isolation, our study started with the isolation of *Vibrio* strains from the diseased shrimp within the shrimp-farmed pond. Furthermore, the pathogenicity of the five *Vibrio* sp. strains (Va-F4, Va-F2, Va-F3, Va-F10, and Val-3) was confirmed, respectively (Figure 1; Supplementary Figure S1). Other reported pathogenic *Vibrio* strains may not be able to cause the disease of vibriosis in shrimp in our study, because the shrimp hosts and other environmental factors are likely the key causative factors of vibriosis as well (Mahoney et al., 2010). We observed that strain Va-F3 showed different pathogenic capabilities in causing the shrimp vibriosis in the two challenge experiments (Figures 1, 7), which were performed in April and November 2017, respectively, highlighting that the environmental factors likely play important roles in the development of vibriosis in the shrimp. More importantly, due to the fact that phages usually are highly specific to infect their hosts (Schmelcher and Loessner, 2014; Kilcher et al., 2018), using the well-known *Vibrio* strains reported in other literature may probably lead to failure of phage therapy in practical applications.

The fast emergence of phage-resistant bacterial variants after treatment with single phage limited the therapeutic applications of phage therapy. Phage cocktail that can delay the appearance of resistant variants has been widely used in practical applications (Gu et al., 2012). Currently, no criteria have been developed to select phages for phage cocktail preparation due to the limited availability and the great diversity of phages. In our previous study, a phage cocktail containing only two phages with divergent genomes showed higher antimicrobial activity than other cocktails and any single phage (Chen et al., 2018). The divergent genomes of the selected phages imply that heterogeneous infection mechanisms against their hosts could be employed by the phages. Thus, the high performance of the phage cocktail can be explained by the fact that the heterogeneous mechanisms likely lead to phage synergy in killing the hosts. Accordingly, in this study, three criteria (see in the section “Materials and Methods”) were set up to select phages for phage cocktail preparation. These criteria warrant that the phages we selected are different in genome and infection mechanism. We selected five phages for cocktail preparation in terms of the observation that the five phages have broad host ranges against *Vibrio* strains (Figure 2; Supplementary Table S1). Our results indicate that the five phages have different host ranges, phylogenies, and divergent genome sequences. These observations suggest that the phages are likely to employ different mechanisms in infecting their hosts. Therefore, although it was shown that two phages (ValLY-3 and VpaJT-1) can infect all five tested pathogenic *Vibrio* strains, including Va-F4, Va-F2, Va-F3, Va-F10, and Val-3 (Figure 2), in our study, the main purpose of including five phages for phage cocktail preparation is to cover a high chance of emergence of phage resistant variants of the *Vibrio* strain during and before the treatment. As shown in Figure 6, bacterial variants emerged rapidly when treated with one single phage, while phage cocktail including the five phages can inhibit the growth of the strain for a long time. Figure 6 also showed the frequencies of phage-resistant variants in *Vibrio* sp. strain Va-F3 when treated with single phage and cocktail. According to the growth curves of Va-F3 treated with different phages, the number of ValLY-3-resistant variants of Va-F3 is similar to that of VspDsh-1 but higher than those of VspSw-1, VpaJT-1, ValSw4-1, and the cocktail (the frequencies of ValLY-3 = VspDsh-1 > VspSw-1 > VpaJT-1 > ValSw4-1). Thus, in our study, the cocktail using the five phages that likely have different mechanisms to infect Va-F3 can kill not only the five isolated pathogenic *Vibrio* strains but also phage-resistant Va-F3 variants. This can be observed from Figure 7 that a satisfying performance in controlling the pathogen growth was obtained in the practical application.

The concept of phage MOI describes the ratio of phages to their host bacteria (Abedon, 2016). The MOI value of 10 was directly used in our *in vitro* experiment. It is difficult to estimate the actual number of a specific bacterial pathogen in a given environment before the phage therapeutic application. Thus, we did not determine the optimal MOI of the phage cocktail for neither *in vitro* nor *in situ* experiments. We delivered the phage cocktail at the final phage concentration of 2 × 10^7^ PFU/ml against pathogen Va-F3 in the 20 L bucket, resulting in a significantly high survival rate of the shrimp challenged by the pathogen *in situ* (Figure 7). A bacterial cell infected by a lytic phage may release up to several hundreds of progeny phage particles with infection activity for the next round infection (Guo et al., 2019). Thus, a lytic phage may generate enormous progeny phages in a given environment full of its host in a short time. This likely suggests that we do not have to use a large dose of phages in the prevention of shrimp vibriosis in practical applications.

The microbial compositions of the aquatic water, as well as the shrimp intestinal tract, are complex (Fan et al., 2019). We observed that the survival rate of the shrimp challenged by the pathogen increased significantly when the phage cocktail was administered as compared to that of the pathogen-challenged shrimp without any treatment. Nevertheless, we did not completely understand what happened to the microbial ecosystems of the aquatic water and the shrimp gut when the phages were administrated. In terms of the predator-prey relationship between phage and its bacterial host, both the environmental microbiota and the shrimp gut microbiota can be interfered by the phage treatment in the experiment *in situ*. We noticed that the intestine of the shrimp (group II) infected by Va-F3 turned to be slightly red, the body was soft, the size of the hepatopancreas was enlarged, and the color of the hepatopancreas was light yellow (Supplementary Figures S3A–D). However, the shrimp of group V with the treatment of the phage cocktail after *Vibrio* challenge did not show significant changes compared to those of group II. Further studies are needed to determine the interplays between phages, microbes, and the shrimp intestine throughout the whole process of phage therapeutic application using metagenomic sequencing, which will shed deep insights into the dynamics of shrimp gut microbiota, provide useful clues toward potential therapeutic applications of phages in aquaculture, and further warrant the success of applications in prevention and treatment of the bacterial infection diseases of the shrimp, and even for other animals.

In conclusion, our study describes a successful example of developing a phage cocktail and applying it in treating *Vibrio* sp. Va-F3 infection disease of the shrimp. Since the use of various antibiotics in aquaculture tends to be banned, the phage cocktail demonstrates the potential of being used as a therapeutic agent for controlling pathogenic bacterial infections, particularly, against vibriosis.

## Data Availability Statement

The datasets generated for this study can be found in the phages were preserved in China Center for Type Culture Collection (Wuhan, China) with CCTCC numbers. M2107548 of ValLY-3, M2107549 of VspDsh-1, M2107547 of VpaJT-1, M2107552 of ValSw4-1, and M2107551 of VspSw-1. The complete genome sequences of the phages have been submitted to the NCBI GenBank database with accession numbers: MH925090 of ValLY-3, MH925091 of ValSw4-1, MH925092 of VpaJT-1, MH925093 of VspDsh-1, and MH925094 of VspSw-1.

## Author Contributions

JF and YM conceived and designed the experiments. JF, LC, TY, QL, SY, HZ, JY, and DD performed the experiments. LC and JF analyzed the data. JY and DD contributed to sample materials. JF, LC, SH, and YM wrote the paper. YM, SH, JF, and LC managed the project.

### Conflict of Interest

The authors declare that the research was conducted in the absence of any commercial or financial relationships that could be construed as a potential conflict of interest.

## References

[ref1] Abedon S. T. (2016). Phage therapy dosing: the problem(s) with multiplicity of infection (MOI). Bacteriophage 6:e1220348. 10.1080/21597081.2016.1220348, PMID: 27738558 PMC5056779

[ref2] Al-Othrubi S. M. Y. Kqueen C. Y. Mirhosseini H. Radu S. Hadi Y. A. (2014). Antibiotic resistance of *Vibrio parahaemolyticus* isolated from cockles and shrimp sea food marketed in Selangor, Malaysia. Clin. Microbiol. 3, 148–154. 10.4172/2327-5073.1000148

[ref3] Bakeraustin C. Stockley L. Rangdale R. Martinezurtaza J. (2010). Environmental occurrence and clinical impact of *Vibrio vulnificus* and *Vibrio parahaemolyticus*: a European perspective. Environ. Microbiol. Rep. 2, 7–18. 10.1111/j.1758-2229.2009.00096.x 23765993

[ref4] Besemer J. Borodovsky M. (2005). GeneMark: web software for gene finding in prokaryotes, eukaryotes and viruses. Nucleic Acids Res. 33, W451–W454. 10.1093/nar/gki487, PMID: 15980510 PMC1160247

[ref5] Biswas B. Adhya S. Washart P. Paul B. Trostel A. N. Powell B.. (2002). Bacteriophage therapy rescues mice bacteremic from a clinical isolate of vancomycin-resistant *Enterococcus faecium*. Infect. Immun. 70, 204–210. 10.1128/IAI.70.1.204-210.2002, PMID: 11748184 PMC127648

[ref6] Chadha P. Katare O. P. Chhibber S. (2016). *In vivo* efficacy of single phage versus phage cocktail in resolving burn wound infection in BALB/c mice. Microb. Pathog. 99, 68–77. 10.1016/j.micpath.2016.08.001, PMID: 27498362

[ref7] Chan B. K. Abedon S. T. Loc-Carrillo C. (2013). Phage cocktails and the future of phage therapy. Future Microbiol. 8, 769–783. 10.2217/fmb.13.47 23701332

[ref8] Chen L. Yang J. Yu J. Yao Z. Sun L. Shen Y. (2005). VFDB: a reference database for bacterial virulence factors. Nucleic Acids Res. 33(Suppl. 1), D325–D328. 10.1093/nar/gki008 15608208 PMC539962

[ref9] Chen L. Yuan S. Liu Q. Mai G. Yang J. Deng D.. (2018). *In vitro* design and evaluation of phage cocktails against *Aeromonas salmonicida*. Front. Microbiol. 9:1476. 10.3389/fmicb.2018.03308, PMID: 30034378 PMC6043867

[ref10] Chhibber S. Kaur S. Kumari S. (2009). Therapeutic potential of bacteriophage in treating *Klebsiella pneumoniae* B5055-mediated lobar pneumonia in mice. J. Med. Microbiol. 57, 1508–1513. 10.1099/jmm.0.2008/002873-0 19018021

[ref11] Crothers-Stomps C. Hoj L. Bourne D. G. Hall M. R. Owens L. (2010). Isolation of lytic bacteriophage against *Vibrio harveyi*. J. Appl. Microbiol. 108, 1744–1750. 10.1111/j.1365-2672.2009.04578.x, PMID: 19886890

[ref12] D’Herelle F. (2011). On an invisible microbe antagonistic to dysentery bacilli. Note by M. F. d’Herelle, presented by M. Roux. Comptes Rendus Academie des Sciences 1917; 165:373–5. Bacteriophage 1, 3–5. 10.4161/bact.1.1.14941

[ref13] Doss J. Culbertson K. Hahn D. Camacho J. Barekzi N. (2017). A review of phage therapy against bacterial pathogens of aquatic and terrestrial organisms. Viruses 9:E50. 10.3390/v9030050, PMID: 28335451 PMC5371805

[ref14] Fan J. Chen L. Mai G. Zhang H. Yang J. Deng D. (2019). Dynamics of the gut microbiota in developmental stages of *Litopenaeus vannamei* reveal its association with body weight. Sci. Rep. 9:734. 10.1038/s41598-018-37042-3 30679786 PMC6345827

[ref15] Frank J. A. Reich C. I. Sharma S. Weisbaum J. S. Wilson B. A. Olsen G. J. (2008). Critical evaluation of two primers commonly used for amplification of bacterial 16S rRNA genes. Appl. Environ. Microbiol. 74, 2461–2470. 10.1128/AEM.02272-07, PMID: 18296538 PMC2293150

[ref16] Frans I. Michiels C. W. Bossier P. Willems K. A. Lievens B. Rediers H. (2011). Vibrio anguillarum as a fish pathogen: virulence factors, diagnosis and prevention. J. Fish Dis. 34, 643–661. 10.1111/j.1365-2761.2011.01279.x, PMID: 21838709

[ref17] Golkar Z. Bagasra O. Pace D. G. (2014). Bacteriophage therapy: a potential solution for the antibiotic resistance crisis. J. Infect. Dev. Ctries. 8, 129–136. 10.3855/jidc.3573, PMID: 24518621

[ref18] Gu J. Liu X. Li Y. Han W. Lei L. Yang Y.. (2012). A method for generation phage cocktail with great therapeutic potential. PLoS One 7:e31698. 10.1371/journal.pone.0031698, PMID: 22396736 PMC3291564

[ref19] Guo Y. Chen P. Lin Z. Wang T. (2019). Characterization of two *Pseudomonas aeruginosa* viruses vB_PaeM_SCUT-S1 and vB_PaeM_SCUT-S2. Viruses 11:318. 10.3390/v11040318, PMID: 30939832 PMC6521218

[ref20] Higuera G. Bastías R. Tsertsvadze G. Romero J. Espejo R. T. (2013). Recently discovered vibrio anguillarum phages can protect against experimentally induced vibriosis in Atlantic salmon, *Salmo salar*. Aquaculture 392, 128–133. 10.1016/j.aquaculture.2013.02.013

[ref21] Kalatzis P. G. Bastías R. Kokkari C. Katharios P. (2016). Isolation and characterization of two lytic bacteriophages, φSt2 and φGrn1; phage therapy application for biological control of *Vibrio alginolyticus* in aquaculture live feeds. PLoS One 11:e0151101. 10.1371/journal.pone.0151101, PMID: 26950336 PMC4780772

[ref22] Kalatzis P. G. Castillo D. Katharios P. Middelboe M. (2018). Bacteriophage interactions with marine pathogenic Vibrios: implications for phage therapy. Antibiotics 7:1. 10.3390/antibiotics7010015 PMC5872126 29495270

[ref23] Karunasagar I. Shivu M. M. Girisha S. K. Krohne G. Karunasagar I. (2007). Biocontrol of pathogens in shrimp hatcheries using bacteriophages. Aquaculture 268, 288–292. 10.1016/j.aquaculture.2007.04.049

[ref24] Kilcher S. Studer P. Muessner C. Klumpp J. Loessner M. J. (2018). Cross-genus rebooting of custom-made, synthetic bacteriophage genomes in L-form bacteria. Proc. Natl. Acad. Sci. USA 115, 567–572. 10.1073/pnas.1714658115, PMID: 29298913 PMC5776983

[ref25] Kokkari C. Sarropoulou E. Bastias R. Mandalakis M. Katharios P. (2018). Isolation and characterization of a novel bacteriophage infecting *Vibrio alginolyticus*. Arch. Microbiol. 200, 707–718. 10.1007/s00203-018-1480-8, PMID: 29372278

[ref26] Lal T. M. Sano M. Hatai K. Ransangan J. (2016). Complete genome sequence of a giant vibrio phage ValKK3 infecting vibrio alginolyticus. Genom. Data 8, 37–38. 10.1016/j.gdata.2016.03.002, PMID: 27114905 PMC4832046

[ref27] Letchumanan V. Chan K. G. Pusparajah P. Saokaew S. Duangjai A. Goh B. H.. (2016). Insights into bacteriophage application in controlling vibrio species. Front. Microbiol. 7:1114. 10.3389/fmicb.2016.01114, PMID: 27486446 PMC4949243

[ref28] Liu W. Lin Y.-R. Lu M.-W. Sung P.-J. Wang W.-H. Lin C.-S. (2014). Genome sequences characterizing five mutations in RNA polymerase and major capsid of phages [greek small letter phi] A318 and [greek small letter phi] As51 of vibrio alginolyticus with different burst efficiencies. BMC Genomics 15:1. 10.1186/1471-2164-15-505, PMID: 24952762 PMC4099482

[ref29] Liu B. Pop M. (2008). ARDB—antibiotic resistance genes database. Nucleic Acids Res. 37(Suppl. 1), D443–D447. 10.1093/nar/gkn656 18832362 PMC2686595

[ref30] Luo R. Liu B. Xie Y. Li Z. Huang W. Yuan J. (2012). SOAPdenovo2: an empirically improved memory-efficient short-read *de novo* assembler. Gigascience 1:18. 10.1186/2047-217x-1-18 23587118 PMC3626529

[ref31] Lv T. Song T. Liu H. Peng R. Jiang X. Zhang W.. (2019). Isolation and characterization of a virulence related *Vibrio alginolyticus* strain Wz11 pathogenic to cuttlefish, *Sepia pharaonis*. Microb. Pathog. 126, 165–171. 10.1016/j.micpath.2018.10.041, PMID: 30391535

[ref32] Mahoney J. C. Gerding M. J. Jones S. H. Whistler C. A. (2010). Comparison of the pathogenic potentials of environmental and clinical *Vibrio parahaemolyticus* strains indicates a role for temperature regulation in virulence. Appl. Environ. Microbiol. 76, 7459–7465. 10.1128/AEM.01450-10, PMID: 20889774 PMC2976215

[ref33] Mateus L. Costa L. Silva Y. Pereira C. Cunha A. Almeida A. (2014). Efficiency of phage cocktails in the inactivation of Vibrio in aquaculture. Aquaculture 424, 167–173. 10.1016/j.aquaculture.2014.01.001

[ref34] Mechri B. Monastiri A. Medhioub A. Medhioub M. N. Aouni M. (2017). Molecular characterization and phylogenetic analysis of highly pathogenic *Vibrio alginolyticus* strains isolated during mortality outbreaks in cultured *Ruditapes decussatus* juvenile. Microb. Pathog. 111, 487–496. 10.1016/j.micpath.2017.09.020, PMID: 28923608

[ref35] Miller A. A. Miller P. F. (2011). Emerging trends in antibacterial discovery: Answering the call to arms. United Kingdom: Horizon Scientific Press.

[ref36] Naville M. Ghuillot-Gaudeffroy A. Marchais A. Gautheret D. (2011). ARNold: a web tool for the prediction of rho-independent transcription terminators. RNA Biol. 8, 11–13. 10.4161/rna.8.1.13346 21282983

[ref37] Oppenheim A. B. Kobiler O. Stavans J. Court D. L. Adhya S. (2005). Switches in bacteriophage lambda development. Annu. Rev. Genet. 39, 409–429. 10.1146/annurev.genet.39.073003.113656, PMID: 16285866

[ref38] Pirnay J. P. De Vos D. Verbeken G. Merabishvili M. Chanishvili N. Vaneechoutte M.. (2011). The phage therapy paradigm: pret-a-porter or sur-mesure? Pharm. Res. 28, 934–937. 10.1007/s11095-010-0313-5, PMID: 21063753

[ref39] Prestridge D. S. (2000). “Computer software of eukaryotic promoter analysis” in Transcription factor protocols. ed. Tymms M. J. (New York: Humana Press), 265–295.

[ref40] Rameshkumar P. Nazar A. K. A. Pradeep M. A. Kalidas C. Jayakumar R. Tamilmani G.. (2017). Isolation and characterization of pathogenic *Vibrio alginolyticus* from sea cage cultured cobia (*Rachycentron canadum* (Linnaeus 1766)) in India. Lett. Appl. Microbiol. 65, 423–430. 10.1111/lam.12800, PMID: 28901019

[ref41] Rao B. M. Lalitha K. V. (2014). Bacteriophages for aquaculture: are they beneficial or inimical. Aquaculture 437, 146–154. 10.1016/j.aquaculture.2014.11.039

[ref42] Rombouts S. Volckaert A. Venneman S. Declercq B. Vandenheuvel D. Allonsius C. N.. (2016). Characterization of novel bacteriophages for biocontrol of bacterial blight in leek caused by *Pseudomonas syringae* pv. porri. Front. Microbiol. 7:279. 10.3389/fmicb.2016.00279, PMID: 27014204 PMC4791379

[ref43] Schattner P. Brooks A. N. Lowe T. M. (2005). The tRNAscan-SE, snoscan and snoGPS web servers for the detection of tRNAs and snoRNAs. Nucleic Acids Res. 33(Suppl. 2), W686–W689. 10.1093/nar/gki366 15980563 PMC1160127

[ref44] Schmelcher M. Loessner M. J. (2014). Application of bacteriophages for detection of foodborne pathogens. Bacteriophage 4:e28137. 10.4161/bact.28137, PMID: 24533229 PMC3919822

[ref45] Shahmuradov I. A. Gammerman A. J. Hancock J. M. Bramley P. M. Solovyev V. V. (2003). PlantProm: a database of plant promoter sequences. Nucleic Acids Res. 31, 114–117. 10.1093/nar/gkg041, PMID: 12519961 PMC165488

[ref46] Sharma A. Gilbert J. A. Lal R. (2016). (Meta) genomic insights into the pathogenome of *Cellulosimicrobium cellulans*. Sci. Rep. 6:25527. 10.1038/srep25527, PMID: 27151933 PMC4858710

[ref47] Song D. Yang Y. Yu B. Zheng B. Deng Z. Lu B.-L.. (2009). Computational prediction of novel non-coding RNAs in *Arabidopsis thaliana*. BMC Bioinf. 10:S36. 10.1186/1471-2105-10-S1-S36, PMID: 19208137 PMC2648795

[ref48] Szczuka E. Kaznowski A. (2004). Typing of clinical and environmental *Aeromonas sp.* strains by random amplified polymorphic DNA PCR, repetitive extragenic palindromic PCR, and enterobacterial repetitive intergenic consensus sequence PCR. J. Clin. Microbiol. 42, 220–228. 10.1128/JCM.42.1.220-228.2004, PMID: 14715756 PMC321687

[ref49] Tamura K. Peterson D. Peterson N. Stecher G. Nei M. Kumar S. (2011). MEGA5: molecular evolutionary genetics analysis using maximum likelihood, evolutionary distance, and maximum parsimony methods. Mol. Biol. Evol. 28, 2731–2739. 10.1093/molbev/msr121, PMID: 21546353 PMC3203626

[ref50] Tendencia E. A. de la Peña L. D. (2001). Antibiotic resistance of bacteria from shrimp ponds. Aquaculture 195, 193–204. 10.1016/S0044-8486(00)00570-6

[ref51] Twort F. W. (1972). An investigation on the nature of ultra-microscopic viruses: the lancet. Acta Orthop. Belg. 38, 566–578.4653800

[ref52] Underwood A. Mulder A. Gharbia S. Green J. (2005). Virulence searcher: a tool for searching raw genome sequences from bacterial genomes for putative virulence factors. Clin. Microbiol. Infect. 11, 770–772. 10.1111/j.1469-0691.2005.01210.x, PMID: 16104996

[ref53] Versalovic J. Koeuth T. Lupski J. R. (1991). Distribution of repetitive DNA sequences in eubacteria and application to fingerprinting of bacterial genomes. Nucleic Acids Res. 19, 6823–6831. 10.1093/nar/19.24.6823, PMID: 1762913 PMC329316

[ref54] Vinod M. G. Shivu M. M. Umesha K. R. Rajeeva B. C. Krohne G. Karunasagar I. (2006). Isolation of *Vibrio harveyi* bacteriophage with a potential for biocontrol of luminous vibriosis in hatchery environments. Aquaculture 255, 117–124. 10.1016/j.aquaculture.2005.12.003

[ref55] Walter W. Sanchez-Cabo F. Ricote M. (2015). GOplot: an R package for visually combining expression data with functional analysis. Bioinformatics 31, 2912–2914. 10.1093/bioinformatics/btv300, PMID: 25964631

[ref56] Wang Y. Barton M. Elliott L. Li X. Abraham S. O’Dea M. (2017). Bacteriophage therapy for the control of *Vibrio harveyi* in greenlip abalone (*Haliotis laevigata*). Aquaculture 473, 251–258. 10.1016/j.aquaculture.2017.01.003

[ref57] Wang R. X. Wang J. Y. Sun Y. C. Yang B. L. Wang A. L. (2015). Antibiotic resistance monitoring in *Vibrio spp.* isolated from rearing environment and intestines of abalone *Haliotis diversicolor*. Mar. Pollut. Bull. 101, 701–706. 10.1016/j.marpolbul.2015.10.027, PMID: 26494250

[ref58] Xu D. Ding W. Ke W. Li F. Zhang P. Guo X. (2018). Modulation of metabolome and bacterial community in whole crop corn silage by inoculating Homofermentative *Lactobacillus plantarum* and Heterofermentative *Lactobacillus buchneri*. Front. Microbiol. 9:3299. 10.3389/fmicb.2018.03299, PMID: 30728817 PMC6352740

[ref59] Yen M. Cairns L. S. Camilli A. (2017). A cocktail of three virulent bacteriophages prevents *Vibrio cholerae* infection in animal models. Nat. Commun. 8:14187. 10.1038/ncomms14187, PMID: 28146150 PMC5296635

[ref60] Young R. Gill J. J. (2015). Phage therapy redux—what is to be done? Science 350, 1163–1164. 10.1126/science.aad6791, PMID: 26785457 PMC6005185

[ref61] Zhang J. Cao Z. Li Z. Wang L. Li H. Wu F. (2015). Effect of bacteriophages on *Vibrio alginolyticus* infection in the sea cucumber, *Apostichopus japonicus* (Selenka). J. World Aquacult. Soc. 46, 149–158. 10.1111/jwas.12177

[ref62] Zhang J. Cao Z. Xu Y. Li X. Li H. Wu F.. (2014). Complete genomic sequence of the *Vibrio alginolyticus* lytic bacteriophage PVA1. Arch. Virol. 159, 3447–3451. 10.1007/s00705-014-2207-z, PMID: 25161033

