# Supplementary material for: Isolation and Characterization of Specific Phages to Prepare a Cocktail Preventing Vibrio sp. Va-F3 Infections in Shrimp (Litopenaeus vannamei)
Source: Front Microbiol. 2019 Oct 11;10:2337. doi: 10.3389/fmicb.2019.02337 (PMC6797625; doi:10.3389/fmicb.2019.02337)
Supplement: Supplementary file 1 [file Data_Sheet_1.PDF]

Title: Isolation and characterization of specific phages to prepare a cocktail preventing *Vibrio* sp. Va-F3 infections in shrimp (*Litopenaeus vannamei*)

Running title: phage cocktail against *Vibrio* sp. Va-F3

Ling Chen[1,2,3#], Jiqiang Fan[1,2,3#], Tingwei Yan[4], Quan Liu[5], Shengjian Yuan[1,2,6], Haoran Zhang[1,2], Jinfang Yang[7], Deng Deng[7], Shuqiang Huang[1,2*], Yingfei Ma[1,2,3*]

[1] Shenzhen Institute of Synthetic Biology, Shenzhen Institutes of Advanced Technology, Chinese Academy of Sciences, Shenzhen, 518055, China.

[2] Key Laboratory of Quantitative Engineering Biology, Shenzhen Institutes of Advanced Technology, Chinese Academy of Sciences, Shenzhen, 518055, China.

[3] Shenzhen Key Laboratory of Synthetic Genomics, Shenzhen Institutes of Advanced Technology, Chinese Academy of Sciences, Shenzhen, 518055, China.

[4] College of Life Science and Technology, Jinan University, Guangzhou, 510632, China

[5] College of Life Science and Oceanography, Shenzhen University, Shenzhen, 518055, China

[6] University of Chinese Academy of Sciences, Beijing 100049, China

[7] R&D Center, Shenzhen Alpha Feed Co., Ltd, Shenzhen, 518000, China

# These authors contributed equally.

*Corresponding to Yingfei Ma, yingfei.ma@siat.ac.cn or shuqiang.huang@siat.ac.cn

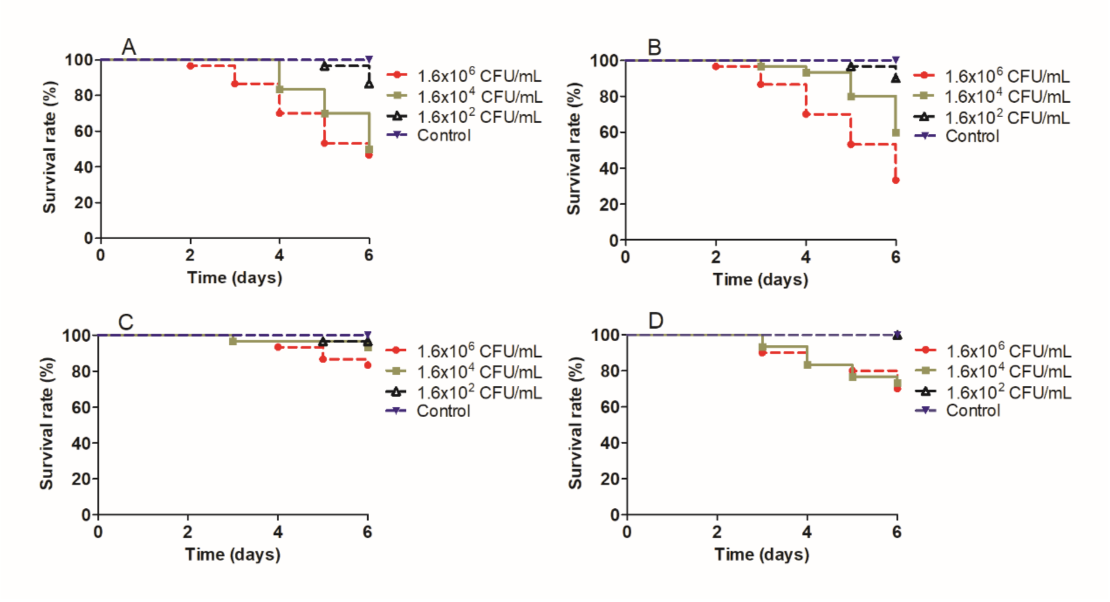

Figure S1 Survival rates of the shrimps infected by the other four vibrio strains *in situ*.

Survival rates of shrimps infected by (A) *Vibrio* sp. Va-F4, (B) *Vibrio* sp. Va-F2, (C) *Vibrio* sp. Va-F10 and (D) *Vibrio* sp. Val-3. As control groups, 500 mL of 2216E medium was included in the cultivating seawater, while for the experimental groups, 500ml of bacterial suspension with three different concentrations at 1.6 $\times 10^2$, 1.6 $\times 10^4$, and 1.6 $\times 10^6$ CFU/mL, respectively.

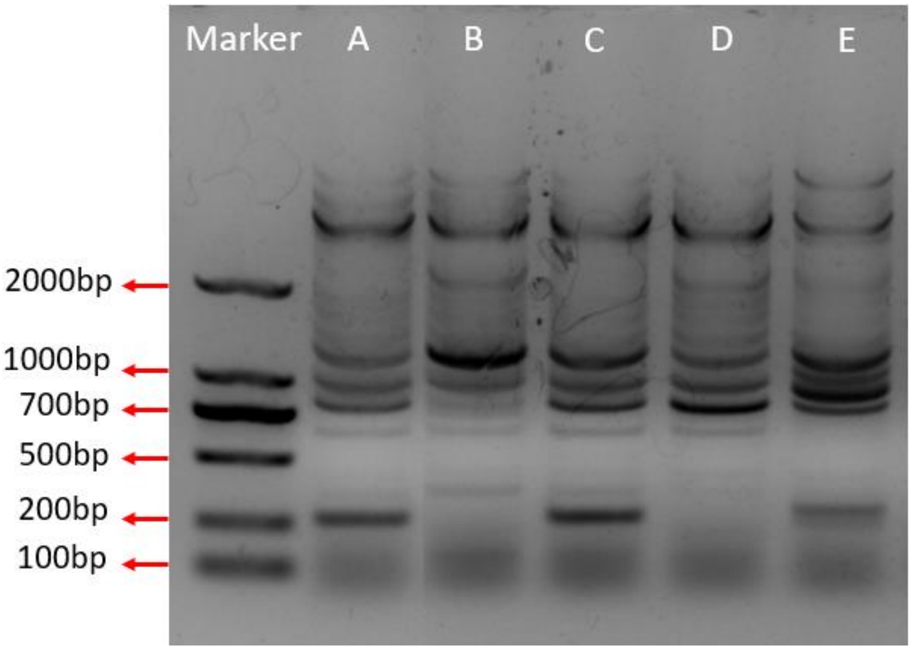

Figure S2. ERIC-PCR products of the five isolated pathogenic *Vibrio* strains. (A) *Vibrio* sp. Val-3 (MH879822), (B) *Vibrio* sp. Va-F4 (MH298559), (C) *Vibrio* sp. Va-F3 (MH298558), (D) *Vibrio* sp. Va-F2 (MH298557), and (E) *Vibrio* sp. Va-F10 (MH298564).

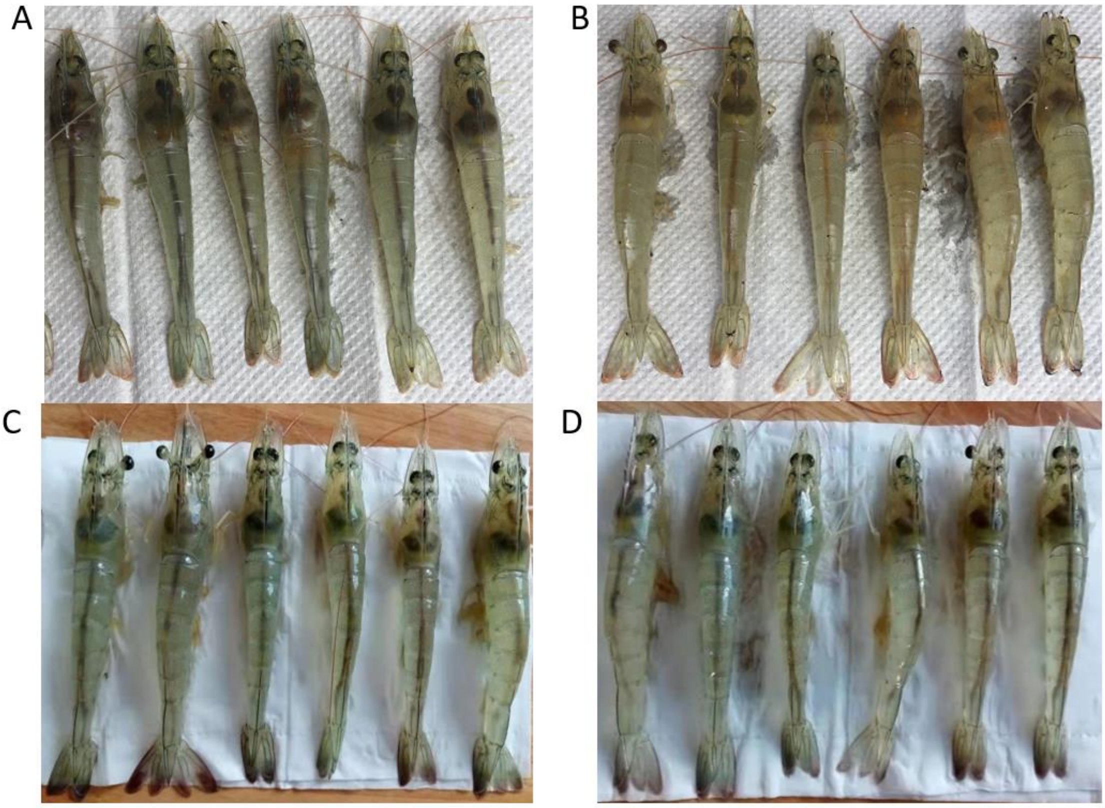

Figure S3 Images of infected/diseased shrimps by *Vibrio* sp. Va-F3. (A)Group V, the shrimp received treatment of the phage cocktail at the final concentration of 2×10^7 PFU/mL after two-day challenge of strain Va-F3 at 2×10^6 CFU/mL; (B) Group II, the shrimp was challenged with Va-F3 at concentration of 2×10^6 CFU/mL; (C) Group IV, the shrimp received treatment of the phage cocktail at the final concentration of 2×10^7 PFU/mL; (D) Group I, the shrimp were treated without adding bacteria, phage cocktail, or any other treatment.

Table S1 information of the strains used in this work

Name	Strain	Source	Mortality	Accession	Blastn	
					Identity	Reference strain / Accesion number
Vibrio sp. Vs-17	Vs-17	Wastewater	< 10%	MH298573	99.8%	*V. owensii* (T); DY05
Vibrio sp. Vp-X11	Vp-X11	Wastewater	< 10%	MH298549	100%	*V. parahaemolyticus* 160807; CP033142.1
Vibrio sp. Vp-4	Vp-4	Shrimp Gut	< 10%	MK377081	99.8%	*V. parahaemolyticus* HH101313; MG386398.1
Vibrio sp. Vp-D9	Vp-D9	Wastewater	< 10%	MH298547	99.8%	*V. parahaemolyticus* msr; MH244245.1
Vibrio sp. Vp-X10	Vp-X10	Wastewater	< 10%	MH298548	99.1%	*V. parahaemolyticus* R13; CP028343.1
Vibrio sp. Vp-D3	Vp-D3	Wastewater	< 10%	MH298543	100%	*V. parahaemolyticus* R3; CP028343.1
Vibrio sp. Vp-D5	Vp-D5	Wastewater	< 10%	MH298545	99.3%	*V.parahaemolyticus* FORC; CP023485.1
Vibrio sp. Vp-F7	Vp-F7	Shrimp Gut	< 10%	MH298562	99.7%	*V. parahaemolyticus* XG409; JQ948037.1
Vibrio sp. Vp-D4	Vp-D4	Shrimp Gut	< 10%	MH298544	100%	*V. parahaemolyticus* VPD14; CP031781.1
Vibrio sp. Vn-19	Vn-19	Wastewater	< 10%	MH298575	99.7%	*V. natriegens* AUCASVE5; JQ277719.1
Vibrio sp. Vn-F1	Vn-F1	Shrimp Gut	66.7%	MH298556	99.7%	*V. natriegens* AUCASCE5; JQ277719.1
Vibrio sp. Vm-6	Vm-6	Wastewater	< 10%	MH298569	100%	*V. metschnikovii* Xmb057; KT986183.1
Vibrio sp. Vm-8	Vm-8	Wastewater	< 10%	MH298570	99.9%	*V. metschnikovii* ST3; KP126899.1
Vibrio sp. Vaz-F9	Vaz-F9	Shrimp Gut	< 10%	MH298563	99.6%	*V. alginolyticus* CAIM 1457; JN603238.1
Vibrio sp. Vaz-15	Vaz-15	Wastewater	< 10%	MH879821	99.7%	*V. alginolyticus* CAIM 1457; JN603238.1
Vibrio sp. Va-F4[b*]	Va-F4	Shrimp Gut	53.4%	MH298559	100%	*V. alginolyticus* b13-1; MK102571.1
Vibrio sp. Va-F2[c*]	Va-F2	Shrimp Gut	66.7%	MH298557	99.7%	*V. alginolyticus* 129; KT224384.1
Vibrio sp. Va-F3[d*]	Va-F3	Shrimp Gut	73.4%	MH298558	99.7%	*V. alginolyticus* CAPL-B-VA1; KX904708.1
Vibrio sp. Va-F10[e*]	Va-F10	Shrimp Gut	16.7%	MH298564	99.8%	*V. alginolyticus* CAPL-B-VA1; KX904708.1

Vibrio sp. Val-3[a*]	Val-3	Wastewater	30.0%	MH879822	99.8%	*V. alginolyticus* B9-1; MK102585.1

A total of 20 strains were tested for the evaluation of host ranges. The 16S rRNA gene sequences of all the strains have been sequenced and submitted to the NCBI with accession as attached above.

a: The host strain used for VspDsh-1 isolation; b: VspSw-1 isolation; c: VpaJT-1 isolation; d: ValLY-3 isolation; e: ValSw4-1 isolation.

*: Verified pathogenic strains in this study.

Shrimp gut: gut of the diseased shrimp; Wastewater: samples collected from the drain exits of shrimp culturing pools
